# Effects of Biochar on the Yield of Melon and the Diversity of Rhizosphere Soil Microbial Communities Under Saline–Alkali Stress

**DOI:** 10.3390/plants14101423

**Published:** 2025-05-09

**Authors:** Yangyang Wang, Qiuyu Lu, Fan Zhang, Wei Wang, Chunyan Wu

**Affiliations:** College of Horticulture, Jilin Agricultural University, Changchun 130118, China; 15948949205@163.com (Y.W.); halutx@163.com (Q.L.); zhangfan061897@163.com (F.Z.)

**Keywords:** biochar, melon, yield quality, soil physicochemical properties, microbial community structure

## Abstract

In this study, the melon variety ‘Da Shetou’ was used as the test material, and pot cultivation was employed with soil collected from Da’an City to investigate the effects of biochar addition on melon yield and quality, rhizosphere soil physicochemical properties, and soil microbial community. The experiment was set up with five treatments: saline–alkali soil (B0), 1% biochar and 99% saline–alkali soil (B1), 3% biochar and 97% saline–alkali soil (B3), 5% biochar and 95% saline–alkali soil (B5), and 7% biochar and 93% saline–alkali soil (B7). This study found that the addition of 3% biochar increased the fruit yield of melons. Compared to the control, the soil bulk density was reduced by 4.99%, 8.66%, 1.77%, and 7.71% under the 1%, 3%, 5%, and 7% biochar treatments, respectively. Biochar addition increased organic matter, alkaline-hydrolyzable nitrogen, available phosphorus, and available potassium concentrations in the rhizosphere soil. Additionally, the total nitrogen, salt concentration, and exchangeable sodium percentage were also reduced. Compared to the B0 treatment, the concentrations of K^+^, Ca^2+^, and Mg^2+^ increased to varying degrees across different treatments, while the concentrations of Na^+^ and Cl^−^ decreased. The relative abundance of dominant bacterial phyla in the soil varied across different treatments. The dominant bacterial phyla included *Proteobacteria*, *Actinobacteriota*, *Acidobacteriota,* and a total of 10 others. The dominant fungal phyla included *Ascomycota*, *Basidiomycota*, *Mortierellomycota,* and a total of seven others. Redundancy analysis (RDA) identified key drivers. Available potassium in the rhizosphere soil of melons was the dominant factor influencing bacterial community composition at the phylum level. Soil bulk density, exchangeable sodium percentage, and total nitrogen were identified as the dominant factors influencing fungal community composition at the phylum level. This study confirmed that 3% biochar application synergistically regulated nutrient cycling and microbial functional groups, thereby enhancing yield of thin-skinned melons (yield increase: 45.22%).

## 1. Introduction

Melon (*Cucumis melo* L.) is a globally significant high-efficiency crop [1]. Advances in horticultural techniques have enabled year-round cultivation, driven by its short cycle and high profitability. However, intensive farming practices (e.g., high multiple cropping indices and excessive agrochemical use) induce soil salinization/alkalization, inhibiting melon growth, reducing yield and quality, and incurring economic losses [2,3].

Soil salinization, as a form of abiotic stress in the 21st century, has a widespread impact globally [4]. Currently, the total area of saline–alkali land in China is approximately 9.91 × 10^7^ hm^2^, with the Songnen Plain having the largest share. Most of the saline soils in the Songnen Plain are soda alkali soils, and Da’an City, located in the western part of the Songnen Plain, is a typical area severely affected by salinization. The soil distribution in this region is highly concentrated, with the city’s unused land area amounting to 1.18 × 10^5^ hm^2^, of which saline–alkali land accounts for more than half [5]. Soil salinity is mainly composed of Na^+^, K^+^, Ca^2+^, Mg^2+^, and Cl^−^. When the concentration of these ions reaches a threshold value that endangers the normal growth of plants, it will cause soil salinization. Saline–alkali soil contains a large amount of salt, which makes it difficult for plants to grow. Soil salinization will cause plant physiological water shortage, affect crop growth and development, and cause large-scale reduction or even no production [6].

In the comprehensive treatment measures of saline–alkali soil, the chemical improvement method is an important technical means. Common modifiers include acidic substances, calcium-containing compounds, and organic modifiers. The application of organic amendments is considered to be one of the most effective improvement methods [7].

Organic amendments, such as biochar, peat, and furfural residue, are increasingly being applied in the practice of ameliorating saline–alkali soils. Biochar has emerged as a hot topic in soil science and related disciplines in recent years. It is a carbon-rich, stable, and highly aromatic solid material formed through the pyrolysis of biomass feedstocks (such as wood, straw, and manure) under complete or partial anaerobic conditions. Biochar boasts superior physicochemical properties, including a large surface area and a surface that is rich in functional groups [8]. Long-term practices have shown that melons grown in saline–alkali soils exhibit a richer sweetness and better taste compared to those cultivated in ordinary soils. Therefore, utilizing biochar to ameliorate heavily saline–alkali soils, optimizing their physicochemical properties, and thereby enhancing the cultivation quality of melons has become a cutting-edge agricultural practice. This method not only helps improve soil structure, but also enhances its water and nutrient retention capabilities, creating a more favorable environment for melon growth and ultimately leading to significant improvement in fruit yield and quality [9]. In recent years, research teams both domestically and internationally have conducted a series of studies on the application of biochar in soil amelioration. Biochar has been shown to increase the field water-holding capacity of soil [10]. Simultaneously, it significantly alleviates soil alkalinity by reducing soil salinity, electrical conductivity, and the concentration of harmful ions such as Na^+^ and Cl^−^ [11]. Particularly, straw biochar effectively improves the soil exchangeable sodium percentage (ESP) by reducing the concentration of soluble Cl^−^ and exchangeable Na^+^, thereby ameliorating soil alkalinity. It also enhances the concentration of available potassium in saline–alkali soils, providing essential nutritional support for plant growth and further strengthening plant stress resistance and productivity [12].

Currently, extensive research both domestically and internationally has also discovered that different types of biochar can influence the growth and development of horticultural crops by altering the abundance and activity of microorganisms. Most studies indicate that biochar can alter soluble solids in tomatoes [13,14]; it also affects organic acids, soluble proteins, and soluble sugars, among other quality attributes of yield in vegetable crops such as celery [15], cucumbers [16,17], and onions [18]. The response of soil microbial communities to biochar depends on the properties of the soil as well as the response variables considered (microbial biomass, richness, and diversity) [19]. The soil microbial community serves as an important indicator for measuring soil health status. These microorganisms are active in a series of crucial biogeochemical cycles, which are essential for maintaining the vitality and productivity of the soil [20]. Therefore, in-depth research and understanding of the changes in the structure and function of the soil microbial community caused by soil management measures, such as the application of biochar, have significant scientific value and practical significance for guiding sustainable agricultural practices and the protection of soil ecosystems. By revealing the response mechanisms and adaptation strategies of these microscopic organisms, we can more effectively regulate the soil environment, promote the improvement of soil quality, and achieve the sustainable development of agriculture [21].

The existing biochar research mainly focuses on the main field crops in saline–alkali soil remediation, while this study pioneered the application of biochar to melon planting under saline–alkali stress. Based on the extensive application of biochar in saline–alkali soil improvement and its potential regulatory effect on soil microorganisms, this study proposes the following hypothesis: adding different proportions of biochar to saline–alkali soil will significantly alleviate the saline–alkali stress response of melon by reducing soil salinity and pH, increasing organic matter concentration and microbial abundance, and promoting yield and quality formation. The 3% biochar rate may achieve the optimal melon–soil synergistic effect by balancing soil improvement effect and cost-effectiveness. This hypothesis was verified by pot experiments combined with high-throughput sequencing analysis.

## 2. Results

### 2.1. Effects of Different Biochar Application Rates on the Physicochemical Properties of Saline–Alkali Soil

#### 2.1.1. Effects of Different Biochar Application Rates on the Chemical Properties of Saline–Alkali Soil

**Figure 1:**
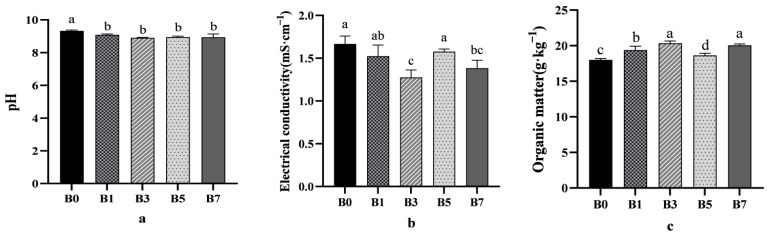

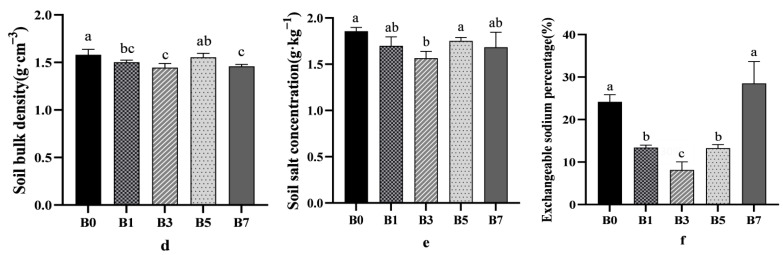
Effect of different proportions of biochar on chemical properties of saline–alkali soils ((**a**) pH; (**b**) electrical conductivity; (**c**) organic matter; (**d**) volume weight of soil; (**e**) salt concentration; (**f**) exchangeable sodium percentage.). Different letters show significant difference (*p* < 0.05).

With the increase of biochar application rates, the pH values of B1, B3, B5, and B7 decreased compared with B0, and B3 decreased by 4.5% compared with B0 (Figure 1a). In addition, B3 treatment reduced the electrical conductivity of melon rhizosphere soil by 13.60% compared with CK (Figure 1b).

Significant variations in organic matter and soil bulk density were observed among different treatments. The B3 and B7 treatments increased the organic matter concentration in the melon rhizosphere saline–alkali soil, showing increases of 12.93% and 11.33%, respectively, compared to the control (B0) (Figure 1c). Additionally, all treatments (B1, B3, B5, and B7) reduced the soil bulk density of the melon rhizosphere saline–alkali soil. Compared to B0, the reductions were 5.00%, 8.66%, 1.77%, and 7.71%, respectively. Although the B5 treatment did not show a statistically significant effect, it still exhibited a decrease in soil bulk density (Figure 1d).

With the increase of biochar application rate, the salt concentration of different treatment groups showed a downward trend. Among them, B3 had a relatively large downward trend (Figure 1e). The order of ESP concentration among treatments was B7 > B0 > B5 > B1 > B3 (Figure 1f).

#### 2.1.2. Effects of Different Biochar Application Rates on Nutrient Availability in Melon Rhizosphere Saline–Alkali Soil

**Figure 2:**
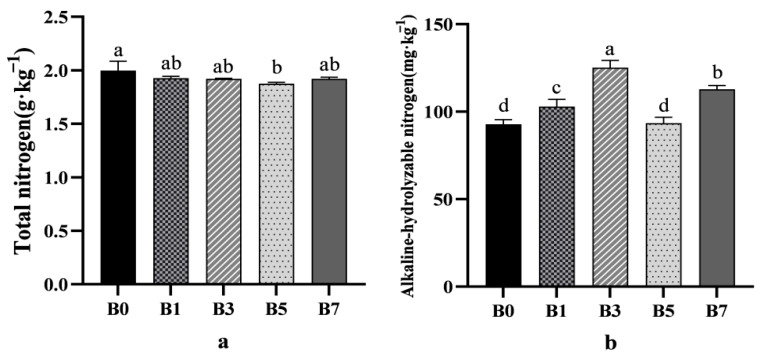

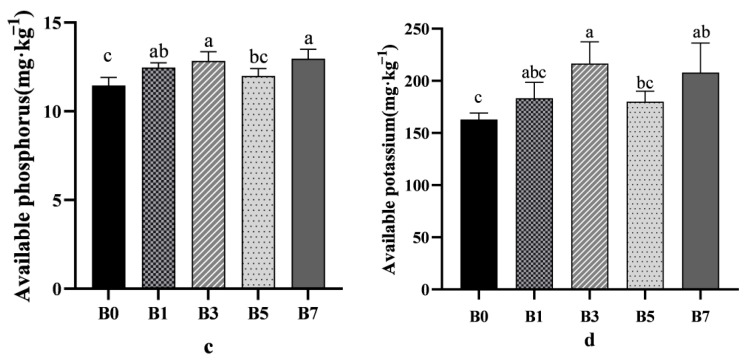
Effect of different rates of biochar on melon rhizosphere saline–alkali soil nutrient availability ((**a**) total nitrogen; (**b**) alkaline-hydrolyzable nitrogen; (**c**) available phosphorus; (**d**) available potassium). Different letters show significant difference (*p* < 0.05).

For total nitrogen, the control group (B0) showed a higher concentration compared to other treatment groups (Figure 2a). Regarding alkaline-hydrolyzable nitrogen, the B3 treatment increased its concentration compared to B0, with the improvement following the order B3 > B7 > B1 > B5 > B0 (Figure 2b).

B3 and B7 treatments increased the available phosphorus concentration, while the improvement effect of B1 and B5 treatment groups was not significant. Compared with the B0 control group, the available phosphorus concentration of the B3 and B7 treatment groups increased by 12.04% and 13.09%, respectively (Figure 2c). The change trend of available potassium concentration was similar to that of available phosphorus, and the improvement effect was B3 > B7 > B1 > B5 > B0 (Figure 2d).

#### 2.1.3. Effects of Different Biochar Application Rates on the Soluble Salt Ion Concentration in Melon Rhizosphere Saline–Alkali Soil

As shown in Table 1, compared with B0, all treatments exhibited varying degrees of increase in K^+^, Ca^2+^, and Mg^2+^ concentrations, while Na^+^ and Cl^−^ concentrations decreased. Specifically, the B3 treatment increased K^+^ and Ca^2+^ concentrations by 30.90% and 34.44%, respectively, compared to B0. For Mg^2+^ concentration, significant improvements were observed between B3 and B7 treatments, showing increases of 41.51% and 33.72%, respectively, versus B0.

Furthermore, B3 reduced Na^+^ concentration (6.43~35.02% decrease compared to B0), while other treatments showed non-significant reductions. Regarding Cl^−^ concentration, both B3 and B7 demonstrated significant decreases compared to B0, with the reduction order being B0 > B5 > B1 > B7 > B3.

### 2.2. Effects of Biochar Application Rates on Melon Fruit Yield and Quality

As shown in Table 2, the application of different biochar rates in saline–alkali soil influenced melon fruit quality parameters. Both B3 and B7 treatments reduced the organic acid concentration in melon fruits by 26.05% and 19.33%, respectively, compared with the control (B0). Notably, the B3 treatment resulted in the lowest organic acid concentration among all treatments. Regarding soluble sugar concentration, the B3 treatment demonstrated the most significant enhancement, increasing levels by 48.46%, 20.55%, 21.92%, and 9.82% compared to the B0, B1, B5, and B7 treatments, respectively.

Additionally, the application of biochar at different rates to saline–alkali soil increased the soluble protein and soluble solids concentration in melon fruits. The improvement in soluble protein followed the order B7 > B3 > B5 > B1 > B0. For soluble solids, the concentrations in B1, B3, B5, and B7 increased by 4.43%, 35.36%, 0.72%, and 13.40%, respectively, compared to B0. Moreover, B3 enhanced the single fruit weight, increasing it by 33.53%, 16.06%, 26.20%, and 4.61% compared to B0, B1, B5, and B7, respectively. The improvement in yield followed the order B3 > B7 > B1 > B5 > B0.

### 2.3. Operational Taxonomic Unit Cluster Analysis of Melon Rhizosphere Saline–Alkali Soil Under Different Biochar Application Rates

**Figure 3:**
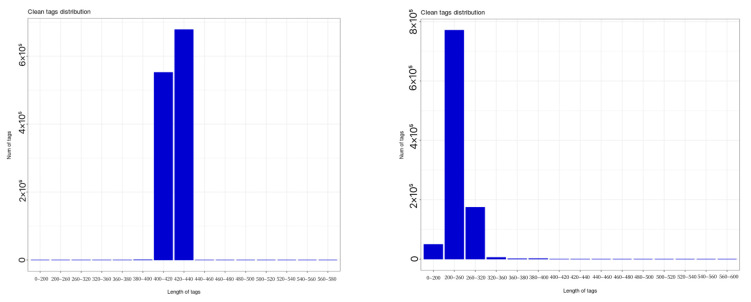
Distribution map of high-quality sample sequences of bacteria (**left**) and fungi (**right**).

As shown in Figure 3, statistical analysis of sequencing data revealed that bacterial 16S rRNA sequencing of soil samples yielded 1,230,942 valid sequences, with lengths primarily concentrated between 400 bp and 440 bp. Clustering at 97% similarity level generated 27,780 bacterial operational taxonomic units (OTUs). Fungal ITS rDNA sequencing produced 1,002,582 valid sequences, mainly ranging from 0 to 320 bp in length, yielding 6678 fungal OTUs at a 97% similarity threshold.

**Figure 4:**
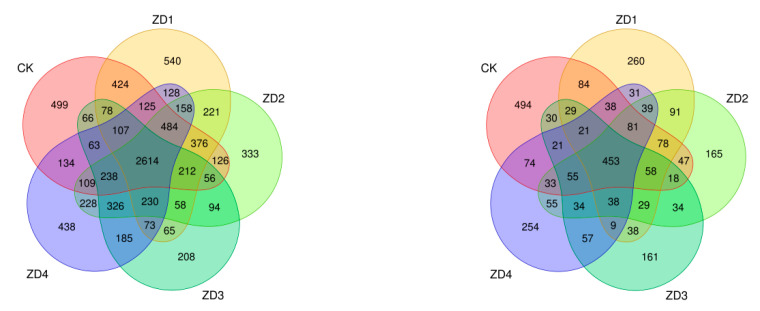
Venn diagram of OTUs of bacteria (**left**) and fungi (**right**).

Figure 4 demonstrates that the OTU numbers for rhizosphere soil bacteria in the B0, B1, B3, B5, and B7 treatment groups were 5711, 5893, 5863, 4673, and 5640, respectively, while the corresponding fungal OTU numbers were 1614, 1377, 1309, 1085, and 1293, respectively. The five rhizosphere soil samples shared 2614 bacterial OTUs (accounting for 9.41% of total) and 453 fungal OTUs (representing 6.78% of total).

### 2.4. Analysis of Microbial Diversity in Melon Rhizosphere Saline–Alkali Soil Under Different Biochar Application Rates

As shown in Table 3, among the five soil samples, B3 rhizosphere soil exhibited the highest number of detected species and bacterial community richness, with the following descending order: B3 > B1 > B0 > B7 > B5. The Shannon index followed the order B1 > B0 > B3 > B7 > B5, while B7 rhizosphere soil showed the lowest Simpson index.

For soil fungi, no significant differences were observed in observed species index, Chao1 index, Simpson index, or Shannon index among the five treatments, though all values were generally higher than those in B5 rhizosphere soil. The Simpson and Shannon indices showed minimal but notable decreases in both B3 and B5 rhizosphere soils, with B7 demonstrating the lowest values.

In summary, B3 rhizosphere soil had the most pronounced effect on bacterial species count and community richness, while all treatments resulted in some reduction of fungal diversity indices.

### 2.5. Analysis of Microbial Community Composition in Melon Rhizosphere Saline–Alkali Soil Under Different Biochar Application Rates

#### 2.5.1. Bacterial Community Composition

**Figure 5:**
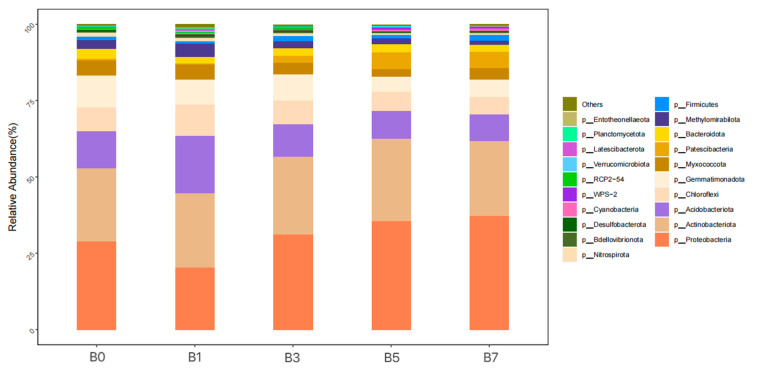
Relative abundance of bacterial communities at the phylum level.

As shown in Figure 5, at the phylum level, bacterial taxa with an average relative abundance ≥ 1% included *Proteobacteria* (20.51~37.38%), *Actinobacteriota* (24.05~26.80%), *Acidobacteriota* (8.59~18.88%), *Chloroflexi* (5.77~10.13%), *Gemmatimonadota* (5.61~10.36%), *Myxococcota* (2.58~5.09%), *Patescibacteria* (0.56~5.46%), *Bacteroidota* (2.12~3.24%), *Methylomirabilota* (1.44~4.19%), and *Firmicutes* (1.16~1.73%). Their average relative abundances were 30.71%, 25.02%, 11.88%, 7.53%, 7.53%, 4.03%, 2.83%, 2.50%, 2.48%, and 1.50%, respectively. The ranking order of relative abundance for each bacterial phylum across treatment groups was *Proteobacteria*: B7 > B5 > B3 > B0 > B1, *Actinobacteriota*: B5 > B3 > B7 > B1 > B0, *Acidobacteriota*: B1 > B0 > B3 > B5 > B7, *Chloroflexi*: B1 > B0 > B3 > B5 > B7, *Gemmatimonadota*: B0 > B3 > B1 > B7 > B5, *Myxococcota*: B0 > B1 > B7 > B3 > B5, *Patescibacteria*: B5 > B7 > B3 > B0 > B1, *Bacteroidota*: B0 > B5 > B3 > B7 > B1, *Methylomirabilota*: B1 > B0 > B3 > B5 > B7, and *Firmicutes*: B7 > B3 > B1 > B5 > B0. *Proteobacteria* and *Firmicutes* showed their highest relative abundance in B7 when compared to B0; *Actinobacteriota* and *Patescibacteria* reached peak abundance in B5; and *Acidobacteriota*, *Chloroflexi,* and *Methylomirabilota* were most dominant in B1. Among all five samples, *Gemmatimonadota*, *Myxococcota,* and *Bacteroidota* exhibited their maximum relative abundance in the B0 treatment group.

#### 2.5.2. Fungal Community Composition

**Figure 6:**
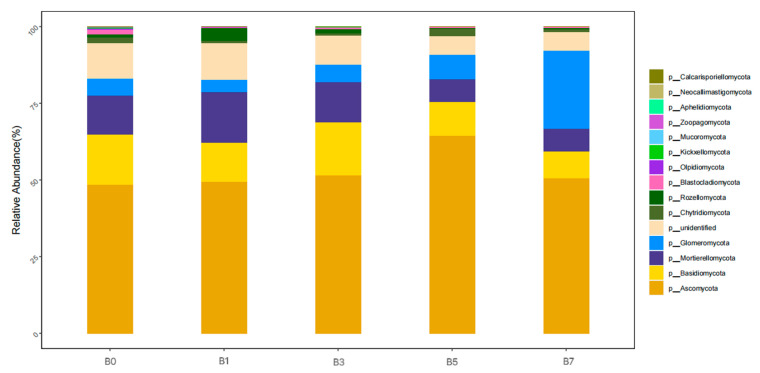
Relative abundance of fungi communities at the phylum level.

As can be seen from Figure 6, at the phylum level, the dominant phyla of fungi in the five soil samples were *Ascomycota* (48.56~64.52%), *Basidiomycota* (8.56~17.43%), *Mortierellomycota* (7.42~16.38%), *Glomeromycota* (4.04~25.29%), *unidentified* (6.65~11.9%), *Chytridiomycota* (0.78~2.54%), and *Rozellomycota* (0.12~4.31%). The average proportions were 52.92%, 13.23%, 11.40%, 9.71%, 9.02%, 1.43%, and 1.41%, respectively.

The relative abundance of *Ascomycota* among different treatments was arranged in the order of B5 > B3 > B7 > B1 > B0, accounting for 64.52%, 51.48%, 50.68%, 49.35%, and 48.56%, respectively. The relative abundances of *Basidiomycota* with treatments B0, B1, B3, B5, and B7 were 16.21%, 12.91%, 17.43%, 11.04%, and 8.56%, respectively, and the order among groups was B3 > B0 > B1 > B5 > B7. Compared with B0, the relative abundances of *Mortierellomycota*, *unidentified,* and *Rozellomycota* were the highest in B1. The relative abundance of *Glomeromycota* varied among different treatments, and the proportion in B7 was the highest. The order of the relative abundance of *Chytridiomycota* among groups was B1 > B3 > B0 > B7 > B5.

### 2.6. Correlation Analysis Between Microbial Community Composition and Soil Physicochemical Properties

**Figure 7:**
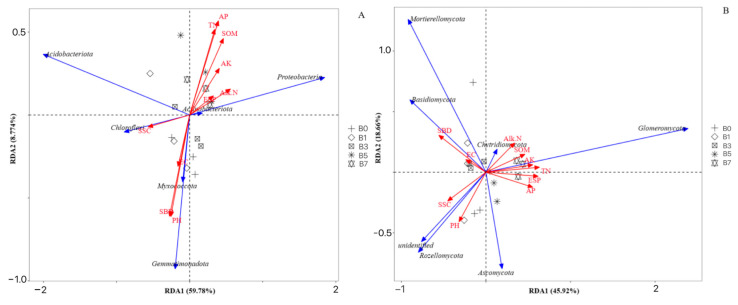
RDA among microbial community structure at the phylum level and soil physicochemical properties ((**A**) bacteria; (**B**) fungi). Note: points represent substrate samples; red arrows represent the physicochemical properties of the substrate; blue arrows represent the substrate microorganisms. When the included angle between influencing factors (between factors and samples) is an acute angle, this indicates a positive correlation between the two factors, and when it is an obtuse angle, this indicates a negative correlation. The longer the ray, the greater the effect of the factor. pH: pH; EC: electrical conductivity; SOM: organic matter; TN: total nitrogen; AP: available phosphorus; AK: available potassium; Alk-N: alkaline-hydrolyzable nitrogen; SSC: salt concentration; SBD: soil bulk density; ESP: exchangeable sodium percentage.

As can be seen from Figure 7, the first ordination axis (59.78%) and the second ordination axis (8.774%) cumulatively explained 68.55% of the variation in the soil bacterial structure. For the RDA analysis of the relationship between fungi and soil physicochemical properties, the first ordination axis (45.92%) and the second ordination axis (18.66%) together explained 64.58% of the species variation, indicating that soil physicochemical properties can well explain the influence on the structure of the soil microbial community.

AP (*p* < 0.05) was the dominant factor driving the changes in the bacterial community at the phylum level; SBD, ESP, and TN (*p* < 0.05) were the dominant factors driving the changes in the fungal community at the phylum level. In the bacterial community, AP was positively correlated with *Proteobacteria*, *Actinobacteriota,* and *Acidobacteriota* and negatively correlated with *Chloroflexi*, *Gemmatimonadota,* and *Myxococcota*. In the fungal community, SBD was positively correlated with *Mortierellomycota* and *Basidiomycota*; ESP and TN were positively correlated with *Glomeromycota*, and negatively correlated with *unidentified* and *Rozellomycota*.

## 3. Discussion

Biochar enhances crop growth by improving soil physicochemical properties and activating rhizosphere microbial activity, thereby improving both crop quality and yield [22]. The effects of corn stalk biochar on plant growth are regulated by multiple factors, including application rate, soil texture, and plant species, demonstrating significant variability. The present study revealed that the 3% biochar treatment yielded optimal results, increasing melon fruit weight and yield while reducing fruit acidity and enhancing sugar concentration. Furthermore, biochar application at different rates effectively improved the soluble solids and soluble protein concentrations in melons. These findings align with numerous studies demonstrating that various types of biochar—either applied alone or in combination with other amendments—can enhance melon quality and yield, including palm leaf biochar [23], biogas slurry–biochar mixtures [24], and biochar derived from anaerobic digestion (AD) byproducts [25].

This study shows that the addition of 3% biochar has a significant regulatory effect on the growth and development of melon, thus effectively improving the fruit quality and increasing the yield. This may be attributed to the fact that it has been shown previously that biochar can effectively increase the concentration of soil nutrients such as alkaline-hydrolyzable nitrogen, available phosphorus, and available potassium, reduce the electrical conductivity, salt concentration, and alkalization degree, improve the composition of soluble salt ions in the soil, and enhance the activity of soil microorganisms in the root zone. As a result, it can improve the soil nutrient supply capacity and the retention efficiency of nutrients in the root layer soil and ultimately increase the yield of melon, the soil utilization efficiency, and the fruit quality. This is consistent with studies conducted by Shwe [12], Tariqul [26], Gong [27], and other researchers.

Zhang [28] et al. believed that biochar could not improve the soil pH of saline–alkali land, and attributed it to the leaching of alkali metal ions and carbonates rich in biochar. However, after the use of biochar in this study, the pH of each treatment decreased, and 3% biochar decreased the pH by 4.50%. This is because the high pH value of saline–alkali soil is related to its ESP. Therefore, as biochar reduces ESP, the pH value will also decrease [29], while improving soil physical and chemical properties and increasing crop yield [30]. During the mature period, the decrease in soil pH value, salt concentration, and alkalization degree mainly promoted the improvement of soil bulk density and the increase of organic matter nutrients [31]. Biochar [30,32] can reduce soil EC and salt concentration. In this study, the addition of 3% and 7% biochar increased the available nutrients in the soil. The mechanism is that, after biochar is added to the soil, it can generate positive and negative charges, effectively adsorbing the nutrients in saline–alkali soil and reducing the leaching loss of nutrients such as alkaline-hydrolyzable nitrogen, available phosphorus, and available potassium in the soil [22]. Compared with the control (B0), the concentrations of K^+^, Ca^2+^, and Mg^2+^ under different treatments increased to varying degrees, while the concentrations of Na^+^ and Cl^−^ decreased. This result is consistent with other studies, indicating that the surface of biochar is rich in functional groups (such as carboxyl groups, hydroxyl groups, etc.), which can adsorb Na^+^ and Cl^−^ in the soil through ion exchange, and at the same time release multivalent cations such as K^+^, Ca^2+^, and Mg^2+^, thus reducing the concentrations of Na^+^ and Cl^−^ [33].

Biochar can improve nutrient availability by reducing the bulk density, alkalinity, and salinity of saline–alkali soil, so as to optimize microbial living environment. As the core driving factor of soil quality, the structure and function of microbial communities will change due to the improvement of biochar, and soil microorganisms themselves have extremely high diversity [34]. In this study, the Alpha diversity analysis of bacteria and fungi in melon rhizosphere soil showed that the number of bacterial species and the richness of bacterial community were the highest in 3% rhizosphere soil, which were 29.75% and 27.33% higher than with the addition of 5% biochar, respectively. At the same time, 1% biochar treatment also increased the Shannon index, reflecting the diversity of the bacterial community, by 1.73% compared with the control group. Kolton [35] and others have shown that the application of biochar increases the richness and diversity of the microbial community. However, with the increase of biochar application rate, the soil fungal diversity index of each treatment group showed a downward trend, and the decrease of 5% treatment was the most significant. The observed species index, Chao1 index, and Shannon index decreased by 24.52%, 19.17%, and 19.94%, respectively, compared with the control group. The results of this study showed that the application of corn straw biochar reduced the OTU number and Alpha diversity index of soil fungal community. This finding is consistent with previous studies on the inhibition of fungal diversity by straw returning [36]. The abundance and diversity of microbial communities depend largely on the pH and nutritional status of the soil. Usually, an increase in pH inhibits microbial growth, resulting in a decrease in community diversity [37]. The soil used in this experiment was saline–alkali soil, and the pH value was as high as 8.91 after adding straw. On the other hand, the carbon–nitrogen ratio (C/N) of rice straw may be relatively large, and the addition of rice straw to the soil leads to an increase in C/N in the soil, and there is not enough nitrogen in the soil to provide fungal activity, thus reducing the diversity of soil fungi.

This study explored the composition and relative abundance of bacterial and fungal community structure in melon rhizosphere saline–alkali soil. The dominant bacterial communities under the five different treatment conditions were concentrated in *Proteobacteria*, *Actinobacteriota*, *Acidobacteriota*, *Chloroflexi*, *Gemmatimonadota*, *Myxococcota*, *Patescibacteria*, *Bacteroidota*, *Methylomirabilota*, and *Firmicutes*. In addition, the dominant fungal flora were all concentrated in *Ascomycota*, *Basidiomycota*, *Mortierellomycota*, *Glomeromycota*, *unidentified*, *Chytridiomycota*, and *Rozellomycota*. At the bacterial phylum level, the richness of *Actinobacteriota* and *Firmicutes* was the lowest in B0, and there were increases to varying degrees with the five treatments. At the fungal phylum level, compared with B0, the abundance of the beneficial fungal phylum *Ascomycota* increased with the increase of biochar. This finding is consistent with prior research [38], where modified biochar not only promotes beneficial bacterial phyla in chrysanthemum-restrictive soil but also enhances fungal phylum abundance, corroborating Zhang [39] et al.’s observations on biochar-mediated microbial regulation. Among these, AP was the predominant factor driving bacterial community variation at the phylum level, whereas SBD, ESP, and TN were the key determinants regulating fungal community shifts at the same taxonomic resolution. AP is negatively correlated with *Chloroflexi*, *Gemmatimonadota,* and *Myxococcota*. The reason may be that the improvement of soil physical and chemical properties caused by the application of biochar promotes the growth of other microbial communities, and there is a certain competitive effect on the growth of *Chloroflexi*, *Gemmatimonadota,* and *Myxococcota* [40]. At the same time, it also confirms that soil physical and chemical properties are the key factors affecting the distribution of bacterial communities in rhizosphere soil [41].

Although this study has gained valuable insights, several limitations still need to be acknowledged. First, pot experiments are carried out under controlled greenhouse conditions, which may not fully reproduce the complexity of the field environment. Secondly, the relatively short experimental period limits evaluation of biochar’s long-term mitigation of soil salinization and maintenance of melon yield. Future research should give priority to field verification of the optimal biochar application rate in different agro-climatic zones. At the same time, long-term field experiments are needed to evaluate the effect of biochar on soil carbon sequestration and the sustainability of salt-tolerant melon variety cultivation.

## 4. Materials and Methods

### 4.1. Saline–Alkali Soil and Biochar

The saline–alkali soil was collected from Da’an City, Jilin Province (from a specific site with defined characteristics). The biochar was derived from corn stover and supplied by Tan Suowei Lai (Guangdong, China) Ecological Environment Technology Co., Ltd.

Physicochemical properties of saline–alkali soil: the EC was 1.86 mS·cm^−1^, the pH was 9.39, the total nitrogen concentration was 2.01 g·kg^−1^, the available phosphorus was 11.57 mg·kg^−1^, and the available potassium was 169.56 mg·kg^−1^.

Physicochemical properties of biochar: the specific surface area was 7.29 m^−2^·g^−1^, the pH was 9.30, the carbon concentration was 45.31%, the phosphorus concentration was 0.243%, the nitrogen concentration was 1.35%, the potassium concentration was 1.18%, and the cation exchange capacity was 24.6 cmol·kg^−1^.

The daytime temperature of the greenhouse was 30~35 °C, while the night-time temperature was 15~18 °C. The relative air humidity was 60~75%.

### 4.2. Experimental Design

#### Plant Material and Treatments

The melon variety used was ‘Da Shetou’ (thin-skinned melon), which is commercially available. The experiment was conducted from March to August 2024 in the greenhouse of the teaching experimental base of the College of Horticulture, Jilin Agricultural University. Potted plants were used, and the specifications of the cultivation pots were a bottom diameter of 17 cm and a top diameter of 23.5 cm. The volume of the pot is 5190 cm^3^. The single-vine pruning method was adopted. A total of five treatments (biochar is applied to the soil in a powdered form and is mixed in the soil and placed evenly in the pot) were applied. There are 15 pots per replicate and a total of three replicates. Sowing and seedling raising were carried out on 29 March, and transplantation was conducted on 28 April. Fruits were retained starting from the 13th node. Artificial pollination began on 26 June, and tags were attached for marking. The harvesting ended on 30 July. The management was the same as the local conventional production management.

### 4.3. Measurement of Yield and Quality Index

After transplantation, three plants with uniform growth vigor were randomly selected and marked from each treatment. The melon fruits were harvested and measured during the mature stage. The measurements were carried out with reference to Zhang [42]. Organic acid concentration: determined by acid-base titration; soluble protein concentration: determined by Coomassie brilliant blue colorimetry; soluble sugar concentration: determined by the anthrone colorimetry method; soluble solids concentration: determined by an Abbe refractometer. The average fruit weight, number of fruits, and yield per plant were calculated to convert the yield per acre.

### 4.4. Soil Sample Collection

Soil samples were collected on 30 July 2024, during the melon ripening stage, with three replicates per treatment. The root-shaking method (the surface soil was removed to the main root distribution layer (usually 0–20 cm depth), and the soil around the main root and lateral root was retained) and ‘S’-shaped random sampling were used to collect rhizosphere soil. The samples were placed into sealed bags and stored in a cooler. Finally, they were transported back to the laboratory and passed through a 2 mm sieve to remove debris. Each sample was divided into two subsamples: one subsample was air-dried naturally at room temperature for physicochemical property analysis, and the other subsample was stored at −80 °C for subsequent DNA extraction and high-throughput sequencing.

#### 4.4.1. Soil Physical and Chemical Properties Testing

Soil physical and chemical determination were performed with reference to Bao [43]. The pH value of the soil was mixed with the soil–water ratio of 1:5 by the potentiometric method; electrical conductivity (mS·cm^−1^) was measured by conductivity meter; the soil bulk density (g·cm^−3^) was measured by the ring knife method; total nitrogen (g·kg^−1^) was determined by the Kjeldahl method; alkaline-hydrolyzable nitrogen (mg·kg^−1^) was determined by the alkali solution diffusion method; available potassium (mg·kg^−1^) was determined using an ammonium acetate extraction-flame photometer; available phosphorus (mg·kg^−1^) was determined by the sodium bicarbonate extraction-spectrophotometer colorimetric method; organic matter was determined by the potassium dichromate volumetric method; salt concentration (g·kg^−1^) was determined using the drying method; the cation exchange capacity (cmol·kg^−1^) was determined by the sodium acetate method; and exchangeable sodium (cmol·kg^−1^) was determined by the NH_4_OAc-NH_4_OH flame photometry method. The exchangeable sodium percentage (ESP) was calculated using the following formula: ESP (%) = (100 × ENa^+^)/CEC.

Determination of water-soluble salt ions in soil: take 50 g of the air-dried soil, add 250 mL of CO^2−^ free distilled water according to a soil–water ratio of 5:1, shake for 5 min, and then use a vacuum filtration machine for filtration. Collect the filtrate for ion determination. Na^+^ (mg·kg^−1^) and K^+^ (mg·kg^−1^) were determined by the flame photometry method; Ca^2+^ (mg·kg^−1^) and Mg^2+^ (mg·kg^−1^) were determined by the EDTA titration method; and Cl^−^ (mg·kg^−1^) was determined by the silver nitrate titration method. The above determinations refer to Bao [43].

#### 4.4.2. Soil Total DNA Extraction and High-Throughput Sequencing

Total genomic DNA was extracted from soil samples using an E.Z.N.A. Soil DNA Kit (Omega Bio-tek, Inc., Norcross, GA, USA). The quality and concentration of the DNA were assessed using a Nanodrop 2000 (Thermo Fisher Scientific, Inc., Waltham, MA, USA). Bacterial primers 338F (5′-ACTCCTACGGGAGGCAGCAG-3′) and 806R (5′-GGACTACNNGGGTATCTAAT-3′) were used to amplify the V3-V4 region of the bacterial 16S rRNA gene, while fungal primers ITS1F (5′-CTTGGTCATTTAGAGGAAGTAA-3′) and ITS2 (5′-TGCGTTCTTCATCGATGC-3′) were employed for PCR amplification of the ITS1-ITS2 region. The PCR products from the same sample were pooled and detected via 1% agarose gel electrophoresis, followed by purification using the Agencourt AMPure XP Nucleic Acid Purification Kit (Beckman Coulter, Bria, CA, USA). After library construction and quality control, paired-end sequencing was performed on the Illumina MiSeq PE300 high-throughput sequencing platform (Illumina Inc., San Diego, CA, USA).

### 4.5. Data Processing and Analysis

The demultiplexed data were separated using QIIME1 (v1.8.0) software, according to the barcode sequences; Pear (v0.9.6) software was used to filter and splice the data, which involved removing scores lower than 20 as well as those containing fuzzy bases and primer mismatch sequences. When splicing, the minimum overlap was set to 10 bp, and the mismatch rate was 0.1. After concatenation, Vsearch (v2.7.1) software was used to remove sequences of less than 230 bp in length, while chimeric sequences were removed using the uchime method based on the Gold Database. The similarity threshold of sequences was 97%. To ensure that the coverage of all samples was fairly high, the data volume of all samples was homogenized to 25,323 sequences. Compared with the Silva128 database using RDP Classifier algorithm, a confidence threshold of 70% was set, and the species classification information corresponding to each OTU was obtained. Based on species annotations and relative abundance results, the species composition histogram was analyzed using R (v3.6.0) software.

Vsearch (v2.7.1) software and the uparse algorithm were used for operational taxonomic unit (OTU) clustering of high-quality sequences. Alpha diversity analysis (including the Shannon, Simpson, and Chao1 indexes) was carried out using QIIME1 (v1.8.0) software. The species composition histogram was analyzed using R (v3.6.0) software. RDA analysis was performed using R (v3.6.0) software based on Weighted UniFrac distance. Statistical significance analysis was carried out using SPSS 27.0 software. The Waller–Duncan test (*p* < 0.05) was used to analyze the single-factor variance data.

## 5. Conclusions

This study confirmed that 3% biochar can be used as the recommended dosage for melon planting in saline–alkali land, which has the functions of increasing yield, improving quality, and improving soil, and is suitable for promotion in similar ecological areas. Its specific application value and core findings are described below.

Compared with the control treatment, adding 3% biochar to the saline–alkali soil of melons has the best effect on yield and quality. However, when 5% biochar is added, compared with adding 3% biochar, the organic acid concentration increases by 29.55%, the soluble protein concentration decreases by 18.56%, the soluble sugar concentration decreases by 17.98%, and the soluble solids concentration decreases by 25.59%, thus inhibiting the improvement of fruit quality. This result provides clear biochar dosage guidance for melon planting in saline–alkali land. Compared with the control, the treatment of adding 3% biochar to the saline–alkali soil of melons has the best effect on improving the physical and chemical properties of the saline–alkali soil and the concentration of soil salinity ions. The treatment of adding 5% biochar reduces the total nitrogen concentration in the soil by 6% compared with B0. This suggests that the application amount should be strictly controlled in practical application to reduce nutrient loss. Furthermore, the observed species index and Chao1 index in the rhizosphere soil of melons treated with 3% biochar are both higher than those of the control treatment. It also increases the relative abundance of beneficial bacteria and fungi and improves the bacterial and fungal communities in the rhizosphere of melons. Redundancy analysis (RDA) shows that AP in the rhizosphere soil of melons is the dominant factor leading to changes in the bacterial community at the phylum level; SBD, ESP, and TN are the dominant factors leading to changes in the fungal community at the phylum level. These findings provide a theoretical basis for regulating microbial communities through biochar to alleviate saline–alkali stress.

In summary, 3% biochar treatment increased the yield and quality of melon, improved the physical and chemical properties and microbial community structure of rhizosphere soil, and was an ideal improvement measure for melon planting in saline–alkali land. Further research: 1. the optimal biochar ratio of different soil types (such as coastal vs. inland saline–alkali soil) needs to be further explored; 2. effects of long-term application on soil health and carbon sequestration; 3. synergistic effect with other modifiers (such as organic fertilizer).

## Figures and Tables

**Table 1:** Effects of different biochar rates on soil ion composition concentrations.

Treatments	K^+^ (mg·kg^−1^)	Na^+^ (mg·kg^−1^)	Ca^2+^ (mg·kg^−1^)	Mg^2+^ (mg·kg^−1^)	Cl^−^ (mg·kg^−1^)
B0	94.04 ± 2.08 ^d^	615.55 ± 2.95 ^a^	193.60 ± 8.21 ^d^	139.52 ± 10.40 ^b^	128.98 ± 5.69 ^a^
B1	105.25 ± 0.67 ^c^	556.22 ± 52.29 ^ab^	209.60 ± 0.92 ^c^	157.44 ± 4.18 ^b^	97.63 ± 8.57 ^c^
B3	123.1 ± 1.67 ^a^	400.00 ± 19.67 ^c^	260.27 ± 6.29 ^a^	197.44 ± 9.01 ^a^	70.41 ± 2.96 ^d^
B5	97.78 ± 2.33 ^d^	576.00 ± 34.06 ^ab^	193.06 ± 2.32 ^d^	156.16 ± 2.31 ^b^	113.60 ± 2.03 ^b^
B7	114.2 ± 2.08 ^b^	496.89 ± 20.00 ^bc^	228.80 ± 1.60 ^b^	186.56 ± 2.24 ^a^	77.51 ± 2.60 ^d^

Note: Different letters in the same column indicate significant differences (*p* < 0.05). Three repeats were used; the same applies below. The data are presented as the mean ± standard error.

**Table 2:** Effects of different biochar rates on yield and quality of melon fruit.

Treatments	Titratable Acid (%)	Soluble Protein (mg·g^−1^)	Soluble Sugar (%)	Dissolved Solid (%)	Single Fruit Weight (g)	Yield (t·ha^−1^)
B0	1.19 ± 0.03 ^a^	0.23 ± 0.02 ^b^	6.48 ± 0.54 ^c^	9.70 ± 0.35 ^b^	240.49 ± 9.23 ^d^	29.19 ± 3.28 ^c^
B1	1.07 ± 0.04 ^b^	0.35 ± 0.05 ^ab^	7.98 ± 0.19 ^b^	10.13 ± 0.46 ^b^	276.69 ± 8.63 ^bc^	36.52 ± 1.14 ^ab^
B3	0.88 ± 0.05 ^c^	0.46 ± 0.02 ^a^	9.62 ± 0.60 ^a^	13.13 ± 0.09 ^a^	321.13 ± 11.49 ^a^	42.39 ± 1.52 ^a^
B5	1.14 ± 0.02 ^ab^	0.37 ± 0.08 ^ab^	7.89 ± 0.20 ^b^	9.77 ± 0.97 ^b^	254.46 ± 9.45 ^cd^	30.75 ± 2.86 ^bc^
B7	0.96 ± 0.01 ^c^	0.50 ± 0.04 ^a^	8.76 ± 0.23 ^ab^	11.00 ± 1.10 ^ab^	306.99 ± 10.90 ^ab^	40.52 ± 1.44 ^a^

Note: Different letters in the same column indicate significant differences (*p* < 0.05). Three repeats were used; the same applies below. The data are presented as the mean ± standard error.

**Table 3:** Effects of biochar application rate on microbial diversity indices in saline–alkali soils.

Microbiome	Sample Name	Observed Species Index	Chao1 Index	Simpson Index	Shannon Index	Coverage Index (%)
Bacteria	B0	4116.60 ± 50.66 ^ab^	4770.27 ± 58.61 ^a^	1.00 ± 0.00 ^a^	9.85 ± 0.08 ^a^	99 ^a^
	B1	4159.93 ± 33.49 ^ab^	4830.87 ± 73.01 ^a^	1.00 ± 0.00 ^a^	10.02 ± 0.00 ^a^	99 ^a^
	B3	4162.30 ± 85.05 ^a^	4847.47 ± 96.37 ^a^	0.99 ± 0.00 ^ab^	9.70 ± 0.11 ^ab^	99 ^a^
	B5	3207.93 ± 42.42 ^c^	3807.16 ± 68.64 ^b^	0.99 ± 0.00 ^b^	8.95 ± 0.15 ^c^	99 ^a^
	B7	3987.90 ± 69.75 ^b^	4686.73 ± 56.21 ^a^	0.99 ± 0.00 ^ab^	9.45 ± 0.14 ^b^	99 ^a^
Fungi	B0	825.00 ± 140.1 ^a^	908.6 ± 161.58 ^a^	0.98 ± 0.01 ^a^	6.87 ± 0.25 ^a^	100 ^a^
	B1	807.67 ± 76.38 ^a^	873.67 ± 76.43 ^a^	0.98 ± 0.00 ^a^	6.75 ± 0.09 ^a^	100 ^a^
	B3	751.33 ± 37.97 ^a^	856.73 ± 39.07 ^a^	0.97 ± 0.01 ^ab^	6.45 ± 0.23 ^a^	100 ^a^
	B5	622.67 ± 8.76 ^b^	734.45 ± 35.69 ^b^	0.90 ± 0.05 ^b^	5.50 ± 0.44 ^b^	100 ^a^
	B7	770.33 ± 52.97 ^a^	839.24 ± 62.57 ^a^	0.97 ± 0.00 ^a^	6.34 ± 0.08 ^a^	100 ^a^

Note: Different letters in the same column indicate significant differences (*p* < 0.05). Three repeats were used; the same applies below. The data are presented as the mean ± standard error.

## Data Availability

The datasets generated during and/or analyzed during the current study are available from the corresponding author on reasonable request. The data are not publicly available due to the priority to complete the follow-up study.
